# Improved Bond Equations for Fiber-Reinforced Polymer Bars in Concrete

**DOI:** 10.3390/ma9090737

**Published:** 2016-08-30

**Authors:** Sadaf Moallemi Pour, M. Shahria Alam, Abbas S. Milani

**Affiliations:** School of Engineering, University of British Columbia, Kelowna, BC V1V1V7, Canada; sadaf.moallemipour@alumni.ubc.ca (S.M.P.); abbas.milani@ubc.ca (A.S.M.)

**Keywords:** fiber reinforced polymer (FRP) rebar, bond strength, factorial design, pullout test

## Abstract

This paper explores a set of new equations to predict the bond strength between fiber reinforced polymer (FRP) rebar and concrete. The proposed equations are based on a comprehensive statistical analysis and existing experimental results in the literature. Namely, the most effective parameters on bond behavior of FRP concrete were first identified by applying a factorial analysis on a part of the available database. Then the database that contains 250 pullout tests were divided into four groups based on the concrete compressive strength and the rebar surface. Afterward, nonlinear regression analysis was performed for each study group in order to determine the bond equations. The results show that the proposed equations can predict bond strengths more accurately compared to the other previously reported models.

## 1. Introduction

Extensive and costly repair and rehabilitation programs are under way in order to extend the service life of reinforced concrete (RC) structures. A major cause of deterioration of RC structures is the corrosion of the steel reinforcement due to exposure to sea water, industrial chemicals, deicing salts, and moisture. This could happen in marine and aggressive environments such as bridges decks, ports, and also pavements. To address this growing concern on durability of concrete structures, fiber reinforced polymer (FRP) bars are gradually becoming popular as an alternative type of reinforcement [1]. These materials consist of fibers embedded in polymeric resin. There are different kinds of fibers and resins that are commonly used in FRPs. Glass FRP (GFRP), carbon FRP (CFRP), and aramid FRP (AFRP) are examples of the most common FRP bars in the current global market of construction. Their resistance to corrosion, non-electromagnetic characteristic, and high strength-to-weight ratio are the main advantages of these types of reinforcements. A wide variety of FRP bars is commercially produced today, going from the simple smooth bars to bars with deformation like ribbed bars and surface treatments to improve bond characteristics [2,3].

The interfacial bond between concrete and reinforcing bars is one of the key aspects in transferring loads from the concrete to the reinforcement with regard to both reinforced and prestressed concrete structures. The resistance against bending, shear, and torsion in reinforced concrete members is closely correlated to their bond behavior. The mechanics of bond stress transfer between concrete and FRP reinforcement has been explored by many researchers [1,2,4,5,6,7,8,9,10,11,12,13,14]. However, due to the lack of well-established standards and different types of commercially available FRP bars and variation in their effective parameters, a plenary model for predicting the bond strength has not been determined yet. 

This paper aims to develop a more comprehensive set of semi-analytical equations on the bond strength between FRP rebar and concrete, with the ultimate goal of predicting a wide range of experimental data reported in the literature [1,2,4,10,11,13,14].

Currently, the existing standards, including the Canadian Code (CSA S806-12) [15] and the Canadian Highway Bridge Design Code (CHBDC 2006) [16], do not provide any specific design models for high strength concrete [4]. Hence, in the present work, the available experimental database has been divided into four groups in a methodical manner. These groups include sand coated and non-sand coated bars embedded in normal and high strength concretes. For each group in the study, an individual predictive equation is suggested and compared to earlier models in the literature. Some design parameters, including the bar surface and location, had not been taken into account in some of the previous models and are included in the new model.

## 2. Experimental Database 

A set of 250 test points formed the experimental database in the current study. They were collected from earlier studies that have been reported during the last 20 years [1,2,4,5,6,7,8,9,10,11,12,13,14]. In all of these experimental studies, the direct pullout test has been employed to measure the bond capacity of the FRP-concrete specimens. The bond strength denotes to the maximum shear resistance per unit contact surface; assuming a uniform bond stress distribution along the embedded length in concrete, the maximum bond strength is defined by the following equation:

(1) τ=Pmaxπdble

where *τ* is the maximum bond strength in MPa, $P_{max}$ is maximum pushout load in N, and $l_{e}$ and $d_{b}$ represent the embedded bar length and the diameter of the bar in mm, respectively. 

The effective variables used during these investigations mostly contain the concrete compressive strength ($f_{c}^{\prime }$), rebar diameter ($d_{b}$), embedment length to bar diameter ratio ($l_{e}/d_{b}$), and the concrete cover to bar diameter ratio ($C/d_{b}$). The selected database includes a wide range of compressive strengths of concrete ($f_{c}^{\prime }$), from 15 to 92 MPa. The ($l_{e}/d_{b}$) and ($C/d_{b}$) ratios vary from 2 to 30 and 2 to 16, respectively. The FRP rebars have several types of surface treatments and deformations: smooth, sand-coated, ribbed, and helically wrapped, among others. A combination of different treatments and deformations has also been applied to some of the bars in the above studies. In order to achieve more accurate models for predicting the bond capacity of different types of FRP rebars, in this study it was decided to allocate the available data into four groups of data. The first and second groups comprise 78 and 48 test results of specimens with non-sand-coated and sand-coated bars, respectively, which have been embedded in *normal* concrete. High strength concrete characteristic ($f_{c}^{\prime }$ > 40 MPa) was used to create the third and fourth groups of data. These two groups include 55 and 71 test results for specimens contained non-sand-coated and sand-coated bars, respectively.

## 3. Suggested Factors Affecting the Bond Behavior of FRP-Reinforced Concrete

For an efficient and reliable transfer of the force between materials in reinforced concrete, the presence of a good interfacial bond is vital. The bond mechanism consists of three parts [17]: (1) mechanical interlocking occurring on the textures on the rebar surface; (2) frictional forces due to roughness of the bar surface and surrounding concrete; and (3) chemical adhesion between the bar and concrete. Beam test and pullout test are the most common types of experiments used by other researches. According to these studies, some of the main parameters that influence the bond behavior include:

1. Concrete compressive strength: the tensile strength of concrete, and hence the bond capacity have a good correlation with the square root of compressive strength of concrete ($\sqrt{f_{c}^{\prime }}$) [18,19].
2. Concrete cover: the concrete affects the bond failure mechanism because it increases the bond strength due to providing the rebar’s confinement [19]. Concrete cover is defined as the distance between the center of embedded reinforcement and the outer surface of the concrete.
3. Bar diameter: the bond capacity is reduced with increased bar diameter [17].
4. Embedment length: the initial bond stiffness of FRP bars increases when the embedment length is long. However, the stress is distributed along a longer length as embedment length increases, therefore there will be a decrease in bond capacity [17].
5. Bar cast position: upward movement of air and water and their getting trapped under the rebar during the horizontal placement of concrete have a negative effect on bond strength [17].
6. Type of fiber: Based on CSA S806-02 [20], AFRP bars show weaker bond behavior than CFRP and GFRP bars. The bond strength of CFRP and GFRP bars can be considered almost the same [17].
7. Type of rebar surface: although in general it has been observed that the deformed bars have better bond behavior than bars with a smooth surface, no definite relation between rebar surface and bond strength has been determined [17].
8. Transverse reinforcement: confinement provided by transverse reinforcement limits the improvement of splitting crack. So, occurrence of bond failure requires a greater force [17].

## 4. Existing Equations for Predicting Bond Strength

For comparative purposes in the present study, the bond models for FRP-concrete have been divided into four groups, with three models based on standard codes including CSA S6-06 [21] (CHBDC 2006) [16], CSA S806-02 [20], and ACI 440-IR-06 [19] (ACI Committee 440 2006), and two models based on recent research works as follows.

Group 1: Some factors such as bar location (*k*_1_), concrete density (*k*_2_), bar size (*k*_3_), bar fiber (*k*_4_), bar surface profile (*k*_5_), have already been considered in the CSA S806 [20] model for FRPs. 

Group 2: In CSA S6 [21], besides the bar location factor, the modulus elasticity of FRP and steel, the area of transverse reinforcement, their spacing, and the flexural strength of concrete (usually $0.4\sqrt{f_{c}^{\prime }})$ have been taken into account [4]. 

Group 3: Based on 151 test specimens comprising concretes with compressive strength varying from 29 to 60 MPa and GFRP bars of 6–19 mm, Okelo and Yuan (2005) [2] suggest an equation for the relationship between bond capacity of GFRPs and concrete compressive strength. 

Group 4: Lee et al. (2008) [1] performed experiments on 54 specimens. The concrete compressive strength (25–92 MPa) and GFRP rebar with sand-coated and helically wrapped external surfaces were used in their experimental program [1]. All the above equations are summarized below.

- Okelo and Yuan (2005) [2]: $\tau_{b}=14.70\frac{\sqrt{f_{c}^{\prime }}}{d_{b}}$
- Lee et al. (2008) [1]: $\tau_{b}=3.3{\left(f_{c}^{\prime }\right)}^{0.3}$
- ACI 440-1R-06 [19]: $\tau_{b}=\left(0.033+0.025\frac{c}{d_{b}}+8.3\frac{d_{b}}{l_{e}}\right)\sqrt{f_{c}^{\prime }}$
- CSA S806-02 [20]: $\tau_{b}=\frac{d_{cs}\sqrt{f_{c}^{\prime }}}{1.15k_{1}k_{2}k_{3}k_{4}k_{5}\pi d_{b}}$
- CSA S6-06 [21]: $\tau_{b}=\frac{0.4d_{cs}\sqrt{f_{c}^{\prime }}}{0.45k_{1}k_{4}\pi d_{b}}$

## 5. A 2^3^ Factorial Design for Parameter Screening

In order to achieve a meaningful set of regression equations to predict the FRP-concrete bond strength, the most important explanatory parameters should be identified/screened first. To this end, the available database was divided into two preliminary groups: specimens with normal and with high strength concrete. Then within each group, and based on earlier experience from published works as reviewed above, two levels (high and low) for three main factors were defined within a 2^3^ factorial design. These factors included: concrete compressive strength ($f_{c}^{\prime }$), rebar diameter ($d_{b}$), and embedment length to bar diameter ratio ($l_{e}/d_{b}$). To reject the null hypothesis during statistical analysis, a significance level of *α* = 0.08 was considered. The data for this screening analysis was chosen specifically from the experimental results of Chaallal (1993) [11] where the high and low levels of the factors are $f_{c}^{\prime }=$ 80 and 30 MPa, $d_{b}=$ 19 and 12.5 mm, and $l_{e}/d_{b}=$ 10 and 5.

**Figure 1:**
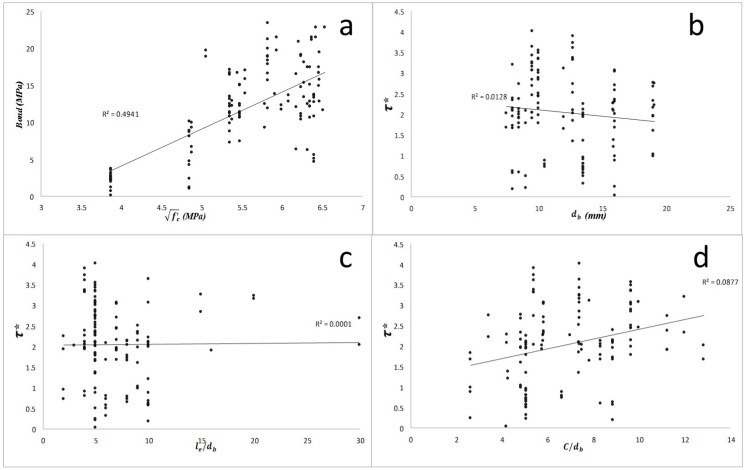
The effect of main factors on the bond strength of normal concrete (study groups 1 and 2): (**a**) square root of concrete compressive strength; (**b**) bar diameter; (**c**) embedment length to bar diameter ratio; (**d**) concrete cover to bar diameter ratio. $\tau^{*}$ is the normalized bond strength.

**Figure 2:**
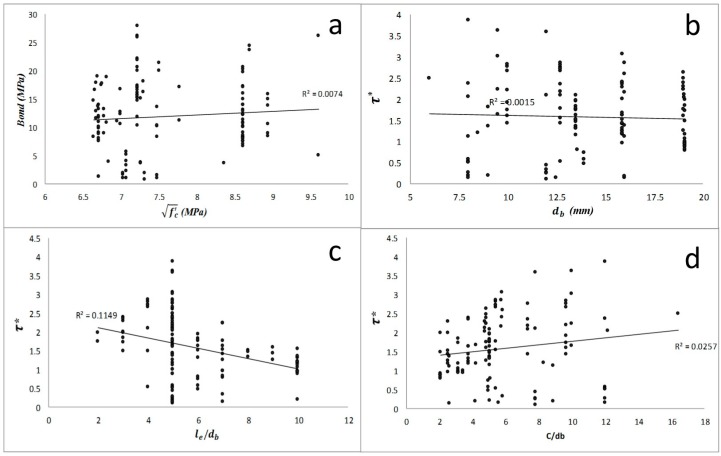
The effect of main factors on the bond strength of high strength concrete (study groups 3 and 4): (**a**) square root of concrete compressive strength; (**b**) bar diameter; (**c**) embedment length to bar diameter ratio; (**d**) concrete cover to bar diameter ratio. $\tau^{*}$ is the normalized bond strength.

**Figure 3:**
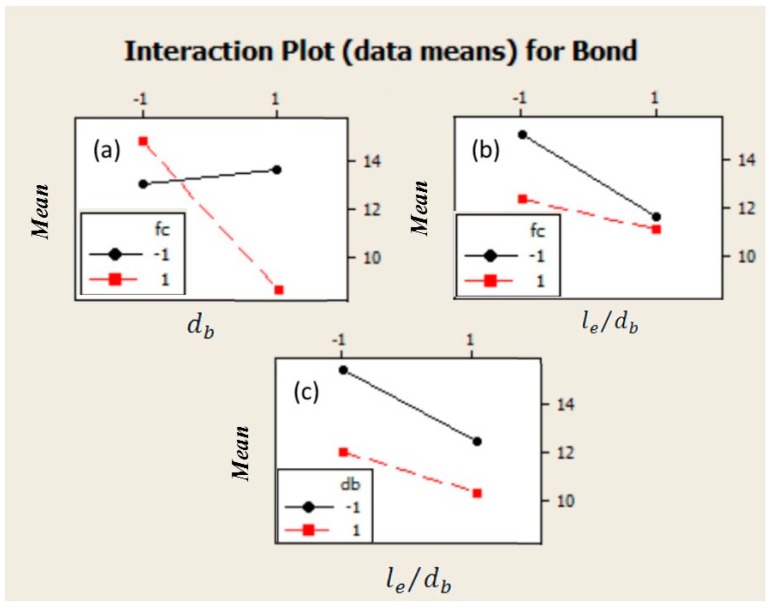
Interaction plots among parameters for experimental bond strength from Chaallal (1993) [11] results. (**a**) Interaction between $f_{c}^{\prime }$ and $d_{b}$; (**b**) Interaction between $f_{c}^{\prime }$ and $l_{e}/d_{b}$; (**c**) Interaction between $d_{b}$ and $l_{e}/d_{b}$.

**Figure 4:**
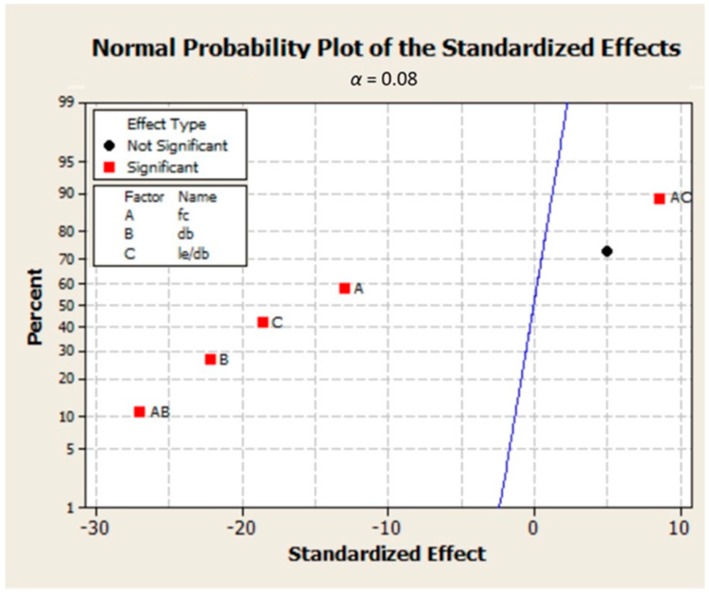
Normal probability plot for identifying significant and nonsignificant factors for Chaallal (1993) [11] results.

As results show in Figure 1 and Figure 2, the bond strength of specimens with normal concrete have a more significant relationship with the three main factors. The *p*-value and percent contribution of each factor and their interactions were also calculated for the chosen data from Chaallal (1993) [11] experimental results (see Table 1). The *p*-value for the parameters $f_{c}^{\prime }\;,\;d_{b},\;l_{e}/d_{b},\;f_{c}^{\prime }d_{b},$ and $f_{c}^{\prime }l_{e}/d_{b}$ were found to be 0.049, 0.029, 0.034, 0.024, and 0.074, respectively, which are all less than 0.08. Similarly, a percent contribution of ~9.6%, 26.8%, 18.8%, 39.6%, and 4% were found for $f_{c}^{\prime }$, $d_{b},\;l_{e}/d_{b},$ $f_{c}^{\prime }d_{b}$, and $f_{c}^{\prime }l_{e}/d_{b}$, respectively. This percentage for all other interactions was nearly zero. According to the above results and other statistical plots of effects in Figure 3 and Figure 4, all the main factors along with two of the interactions should be taken into account in developing subsequent regression equations for bond strength predictions. The almost parallel lines observed in Figure 3c indicate that there is no significant effect of interaction between bar diameter and embedment length to bar diameter ratio on bond strength. Considering the influence of concrete compressive strength, the nonparallel lines observed in Figure 3a,b represent a strong and moderate effect of interaction between this variable and bar diameter and embedment length to bar diameter ratio on bond strength, respectively. The results can also be confirmed by normal plot in Figure 4; the black circle point, which represents the interactions between rebar diameter and embedment length to bar diameter ratio, is close to the normal plot, therefore this interaction does not have a significant effect on output.

## 6. New Bond Prediction Equations

As described earlier, based on the concrete compressive strength and the type of rebar surface, the current databases of 250 specimens were divided into four groups. Eighty percent of the data within each group were randomly selected and used to develop the prediction equation. Then, for validation purposes within each group, the remaining 20% of the data was employed and verified via regression goodness-of-fit measures. According to the above-performed factorial design (screening analysis) and previously published models in the literature, the bond equations may take a general form of:

(2) $$\tau =f\left(f_{c}^{\prime },d_{b},\frac{C}{d_{b}},\frac{l_{e}}{d_{b}},f_{c}^{\prime }\frac{C}{d_{b}},f_{c}^{\prime }\frac{l_{e}}{d_{b}},\frac{f_{c}^{\prime }}{d_{b}}\right)$$

By assuming a linear combination of significant factors and performing the regression analysis in available software such as Minitab, the obtained bond equation for each group is given in Table 2.

## 7. How to Compare Different Bond Models

First, in order to evaluate the performance and accuracy of different models, they may be exclusively applied to each of the four study groups; namely, to compare their predictability on the measured bond strength within each group. As seen in the Section 4, all the earlier equations in the literature contain the concrete compressive strength and bar diameter. However, unlike the equations in the design code guidelines, the equations proposed by Lee et al. (2008) [1] and Okelo and Yuan (2005) [2] do not consider the concrete cover and embedment length. The new models in the present work (Table 1) are checked and compared to these available equations in the next section. In doing so, the selected statistical measures for model comparisons are performance factor (*PF*), the average absolute error (AAE), coefficient of variation (COV), and root mean squared error (*RMSE*) [22]. 

According to Montgomery (2008) [23], the difference between an experimental database and a prediction equation may be best evaluated via the standard deviation performance factor and the coefficient of variation of the performance factor. A proposed model may be said to accurately and correctly predict the outcome only if the average of the *PF* is in the proximity of 1, with a small standard deviation. 

(3) $$\left(PF\right)=\frac{\tau_{test}}{\tau_{calc}}$$

The COV shows the accuracy of a suggested equation by evaluating the ratio of the standard deviation to the mean of performance factor and is defined as:

(4) $$COV\left(\%\right)=\frac{Standard\;deviation\;\left(\sigma \right)}{Mean\;\left(\tau \right)}\times 100$$

To reduce the amount of scatter in predicting the data, the coefficient of variation of a fitted model should decrease. To consider the total error in prediction results, the *AAE* can be evaluated using the following equation:

(5) $$AAE\left(\%\right)=\frac{1}{n}\sum^{\text{​}}\frac{\left|\tau_{test}-\tau_{calc}\right|}{\tau_{test}}\times 100$$

The root mean square error (*RMSE*) is another frequently used measure of the difference between values predicted by a model and those observed from experiments. The *RMSE* of a prediction model with respect to the estimated variable is defined as the square root of the mean squared error as:

(6) $$RMSE=\sqrt{\frac{\sum^{\text{​}}{\left(\tau_{test}-\tau_{calc}\right)}^{2}}{n}}$$

## 8. Results of the Bond Equations for Each Study Group

Results from the descriptive statistical analysis performed on different bond models within each study group are summarized in Table 3, Table 4, Table 5 and Table 6. For the first group, the models by Lee et al. (2008) [1] and Okelo and Yuan (2005) [2] have produced poor prediction results for samples with normal concrete. The average and standard deviation of *PF* and *COV* are larger than the other models. For the groups of specimens with high strength concrete, CSA S6 [21] and CSA S806 [20] models have the least accurate results. In the first group with normal concrete specimens and non-sand-coated rebar, ACI 440-1R-06 [19] and Okelo and Yuan (2005) [2] have the lowest *RMSE*, which is about 6.2. The model suggested by CSA S6 [21] and Lee et al. (2008) [1] have almost the same *AAE* (187% and 193%, respectively) and *RMSE*, which is about 5.7. However, the equation proposed in the present study has the best results of *AAE* = 59%, *RMSE* = 3.84, and, *R*^2^ = 0.62. For the second group of normal concrete with sand-coated bars, ACI 440-1R-06 [19] and CSA S806’s [20] equations have good *RMSE* values of 5.09 and 5.8, and *AAE* values of 30% and 33%, respectively. The new proposed model, however, has the best results of *AAE* = 12% and *RMSE* = 2.34 in this group. According to results, the performances of equations compared to each other in the third group illustrate that CSA S6 [21] and CSA S806 [20] are not suitable for predicting the bond in high strength concrete. In this group, the proposed new model (*RMSE* = 4.69 and *R*^2^ = 0.48) seems the best appropriate equation for specimens with high strength concrete and with non-sand-coated rebar. For the last (fourth) group, which includes the specimens with sand-coated rebar embedded in high strength concrete, the model by Lee et al. (2008) [1] had a good *RMSE* = 5.57, and most of its other statistical parameters are better than previous models. However, the new model is still the best for this group (*AAE* = 27%, *RMSE* = 3.96). 

**Figure 5:**
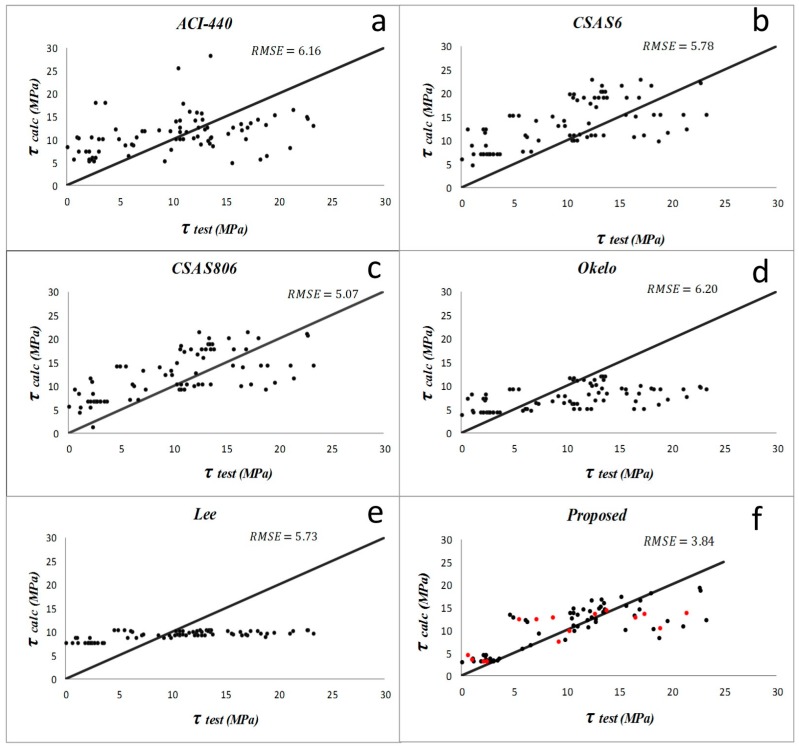
Bond strength prediction results by different models (**a**–**f**) on the normal concrete with non-sand-coated fiber-reinforced polymer (FRP) bars (study group 1). The data used for validation are shown as red points in figure (**f**).

**Figure 6:**
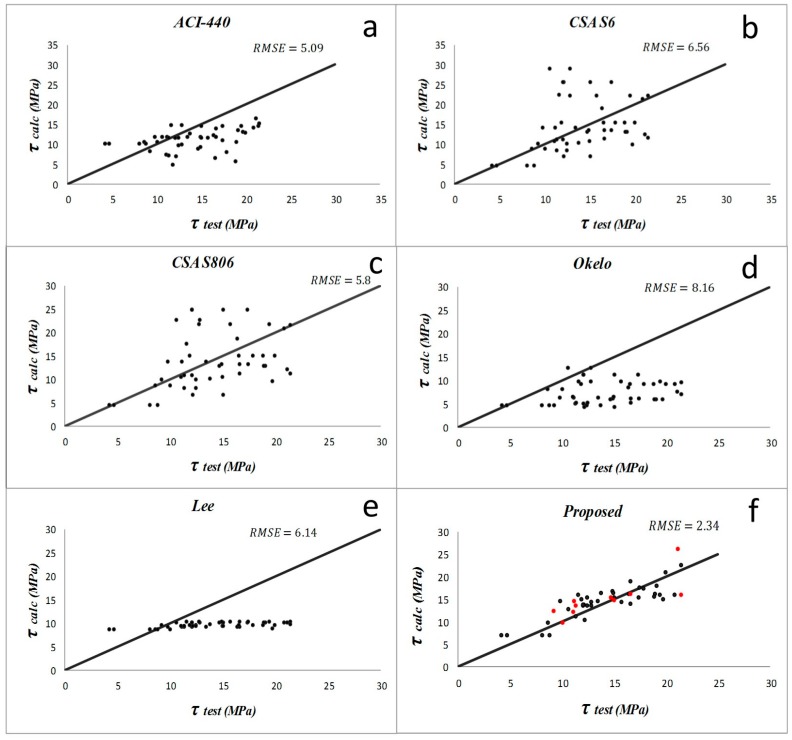
Bond strength prediction results by different models (**a**–**f**) on the normal concrete with sand-coated FRP bars (study group 2). The data used for validation are shown as red points in figure (**f**).

**Figure 7:**
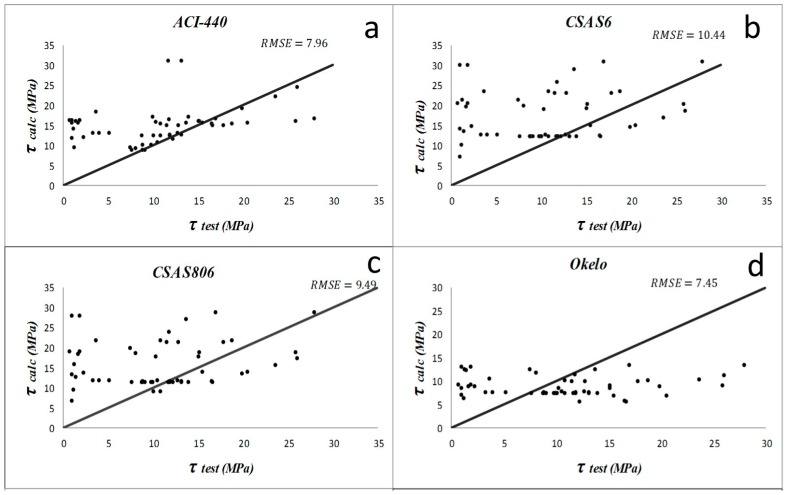

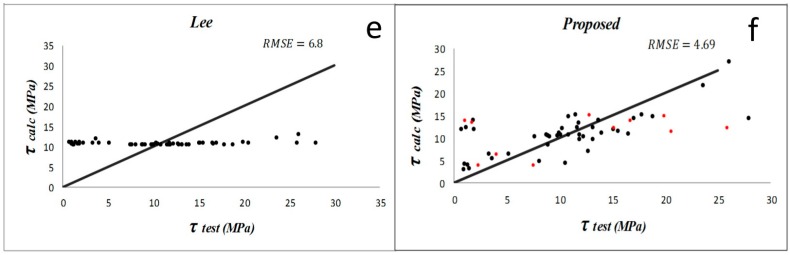
Bond strength prediction results by different models (**a**–**f**) on the concrete with high strength and non-sand-coated FRP bars (study group 3). The data used for validation are shown as red points in figure (**f**).

**Figure 8:**
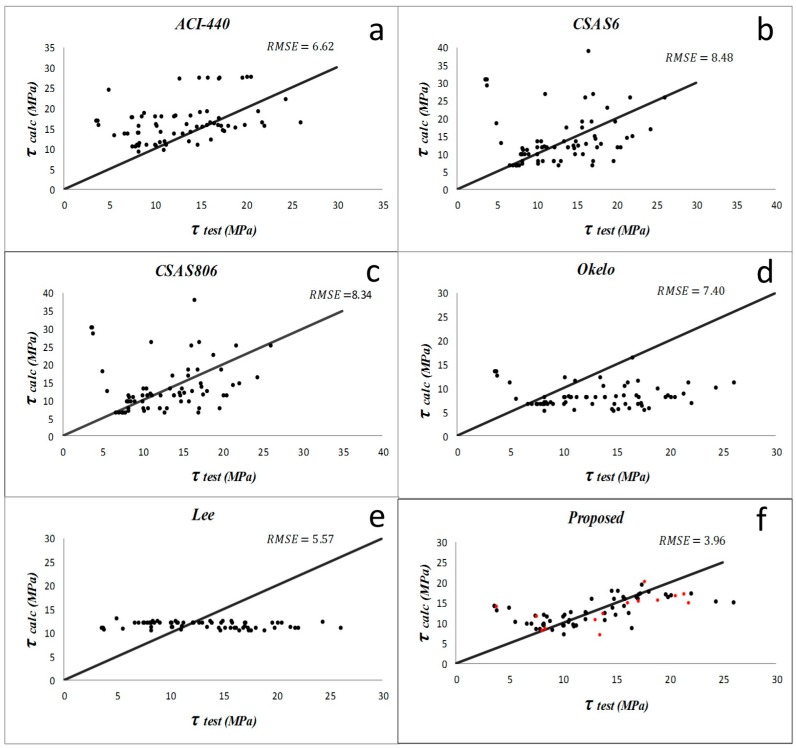
Bond strength prediction results by different models (**a**–**f**) on the concrete with the concrete high strength sand-coated FRP bars (study group 4). The data used for validation are shown as red points in figure (**f**).

The comparisons of the previous bond equations to predict the bond strength of pullout specimens proposed by different researchers and the improved proposed equation are displayed in Figure 5, Figure 6, Figure 7 and Figure 8. ACI and CSA codes can predict the bond strength values accurately because these formulas are based on the experimental data carried out on normal strength concrete along with other factors like bar diameter, concrete cover, and embedment length. However, since concrete compressive strength has an important effect on bond performance, CSA and ACI codes cannot predict the bond strength of rebar in high strength concrete members accurately, whereas Lee et al. (2008) [1] and Okelo and Yuan (2005) [2] models show better prediction as they were based on high strength concrete test results. 

## 9. Conclusions

This study aimed at developing new equations for predicting the bond strength of FRP bars with and without sand coating embedded in normal and high strength concrete. A total of 250 test points were collected from previous reports in the literature in order to obtain a comprehensive model. The results of statistical analysis and comparisons among the previous and proposed models led to the following conclusions:

- According to the conducted factorial design, the square root of compressive strength of concrete ($\sqrt{f_{c}^{\prime }}$), rebar diameter ($d_{b}$), the embedment length to bar diameter ratio ($\frac{l_{e}}{d_{b}}$), and also the concrete cover to bar diameter ratio ($\frac{C}{d_{b}}$) have a good correlation with the bond capacity of FRP to concrete. It can also be concluded that the combined effects of compressive strength of concrete and the other three factors ($\sqrt{f_{c}^{\prime }}/d_{b},\;\sqrt{f_{c}^{\prime }}\times \frac{C}{d_{b}},\;\sqrt{f_{c}^{\prime }}\times \frac{d_{b}}{l_{e}}$) could influence the bond behavior significantly. Hence, considering these nonlinear interaction terms, besides the main factors, significantly improved the accuracy of the bond strength predictions. The highest percent contribution among all these factors was found to be for $\sqrt{f_{c}^{\prime }}/d_{b}$ ratio.
- Based on the comparative study among different bond equations for different groups of concrete specimens, all the statistical measures of the newly proposed equations showed superior performance. In particular, for the tests carried out with normal concrete, the COV of the new bond equations is 17% less than the average *COV* of previous models. Also, the average *PF* is very close to 1 and the *R*^2^ is ~0.6. Therefore, the proposed models seem to be more accurate to predict the bond strength.
- Since most of the previous models are not applicable to high strength concrete members, the presented improved bond equations can be further evaluated and compared to similar new models/new experimental data that will be developed in future.

## Figures and Tables

**Table 1:** Factorial design results for main effects and significant interactions.

Parameters	Sum of Squares	Contribution %	*P* Value
Main Effects			
$f_{c}^{\prime }$	5.53	9.6	0.049
*d_b_*	15.4	26.8	0.029
*l_e_*/*d_b_*	10.8	18.8	0.034
2-Way Interactions			
$f_{c}^{\prime }$ × *d_b_*	22.7	39.6	0.024
$f_{c}^{\prime }$ × *l_e_*/*d_b_*	2.31	4.02	0.074
*d_b_* × *l_e_*/*d_b_*	0.78	1.36	0.126

**Table 2:** The predictive bond equations developed in the present study.

Group Number	Concrete Compressive Strength	Rebar Surface	Bond Equation
1	Normal Concrete	Non-Sand-Coated	τ = −5.3014 + 1.76$\left(\sqrt{f_{c}^{\prime }}\right)$ − 21.058$\left(d_{b}/l_{e}\right)$ + 1.914$\left(C/d_{b}\right)$ − 159.614$\left(1/d_{b}\right)$ + 14.209$\left(\sqrt{f_{c}^{\prime }}/d_{b}\right)$ − 0.0379$\left(C\sqrt{f_{c}^{\prime }}/d_{b}\right)$ + 5.85$\left(d_{b}\sqrt{f_{c}^{\prime }}/l_{e}\right)$
2	Normal Concrete	Sand-Coated	τ = − 78.85 + 14.806$\left(f_{c}^{\prime }\right)$ + 133.97$\left(d_{b}/l_{e}\right)$ + 16.747$\left(C/d_{b}\right)$ − 941.28$\left(1/d_{b}\right)$ + 193.256$\left(\sqrt{f_{c}^{\prime }}/d_{b}\right)$ − 3.187$\left(C\sqrt{f_{c}^{\prime }}/d_{b}\right)$ − 16.695$\left(d_{b}\sqrt{f_{c}^{\prime }}/l_{e}\right)$
3	High Strength Concrete	Non-Sand-Coated	τ =450.77 − 64.15$\left(f_{c}^{\prime }\right)$ − 1371.33$\left(d_{b}/l_{e}\right)$ + 11.237$\left(C/d_{b}\right)$ − 2494.57$\left(1/d_{b}\right)$ +321.312$\left(\sqrt{f_{c}^{\prime }}/d_{b}\right)$ − 1.25$\left(C\sqrt{f_{c}^{\prime }}/d_{b}\right)$ + 205.133$\left(d_{b}\sqrt{f_{c}^{\prime }}/l_{e}\right)$
4	High Strength Concrete	Sand-Coated	τ = 13.013 − 1.515$\left(f_{c}^{\prime }\right)$ + 427.715$\left(d_{b}/l_{e}\right)$ + 11.88$\left(C/d_{b}\right)$ − 1965.237$\left(1/d_{b}\right)$ + 239.92$\left(\sqrt{f_{c}^{\prime }}/d_{b}\right)$ − 1.397$\left(C\sqrt{f_{c}^{\prime }}/d_{b}\right)$ − 45.88$\left(d_{b}\sqrt{f_{c}^{\prime }}/l_{e}\right)$

**Table 3:** Comparison of bond equations for the first group.

Bond Equations	$PF=\tau_{test}/\tau_{calc}$			*AAE* (%)	*RMSE*
	Avg. *PF*	Std. *PF*	*COV* (%)		
Okelo and Yuan (2005) [2]	1.35	0.83	61.47	119.96	6.20
Lee et al. (2008) [1]	1.04	0.60	57.54	193.10	5.73
ACI 440-1R-06 [19]	0.86	0.47	55.04	205.94	6.16
CSA S806-02 [20]	0.79	0.47	59.26	160.64	5.07
CSA S6-06 [21]	0.71	0.41	57.72	187.15	5.78
Proposed bond equation	0.97	0.40	41.37	59.48	3.84

**Table 4:** Comparison of bond equations for the second group.

Bond Equations	$PF=\tau_{test}/\tau_{calc}$			*AAE* (%)	*RMSE*
	Avg. *PF*	Std. *PF*	*COV* (%)		
Okelo and Yuan (2005) [2]	2.11	0.74	35.13	46.12	8.16
Lee et al. (2008) [1]	1.45	0.41	27.79	33.49	6.14
ACI 440-1R-06 [19]	1.35	0.54	39.98	29.94	5.09
CSA S806-02 [20]	1.17	0.45	37.98	32.66	5.80
CSA S6-06 [21]	1.14	0.45	39.09	35.76	6.56
Proposed bond equation	1.00	0.18	18.11	12.30	2.34

**Table 5:** Comparison of bond equations for the third group.

Bond Equations	$PF=\tau_{test}/\tau_{calc}$			*AAE* (%)	*RMSE*
	Avg. *PF*	Std. *PF*	*COV* (%)		
Okelo and Yuan (2005) [2]	1.29	0.84	64.45	168.32	7.45
Lee et al. (2008) [1]	1.00	0.62	62.13	182.26	6.80
ACI 440-1R-06 [19]	0.74	0.42	57.15	246.75	7.96
CSA S806-02 [20]	0.74	0.46	62.15	278.18	9.49
CSA S6-06 [21]	0.68	0.42	62.13	317.08	10.44
Proposed bond equation	0.97	0.5	51.23	118.20	4.69

**Table 6:** Comparison of bond equations for the fourth group.

Bond Equations	$PF=\tau_{test}/\tau_{calc}$			*AAE* (%)	*RMSE*
	Avg. *PF*	Std. *PF*	*COV* (%)		
Okelo and Yuan (2005) [2]	1.65	0.74	44.09	50.86	7.40
Lee et al. (2008) [1]	1.13	0.48	42.71	42.81	5.57
ACI 440-1R-06 [19]	0.82	0.32	38.45	60.08	6.62
CSA S806-02 [20]	1.13	0.51	45.21	69.60	8.34
CSA S6-06 [21]	1.11	0.50	45.21	70.79	8.48
Proposed bond equation	1.00	0.32	31.71	27.04	3.96

## Abbreviations

$f_{c}^{\prime }$	Concrete compressive strength
$d_{b}$	Rebar diameter
$l_{e}/d_{b}$	Embedment length to bar diameter ratio
$C/d_{b}$	Concrete cover to bar diameter ratio
$d_{cs}$	Smallest of the distance from the closest concrete surface to the center of the bar being developed or two-thirds the center-to-center spacing of the bars being developed
$k_{1}$	Bar location
$k_{2}$	Concrete density
$k_{3}$	Bar size
$k_{4}$	Bar fiber
$k_{5}$	Bar surface profile
n	Number of tests
$R^{2}$	Root square
τ	Bond strength
$\tau_{test}$	Experimental bond strength
$\tau_{calc}$	Predicted bond strength
α	Significance level
σ	Standard deviation
RMSE	Root Mean Square Error
AAE	Average absolute error
